# Pulmonary Hypertension in Systemic Lupus Erythematosus: Current Evidence and Future Directions

**DOI:** 10.7759/cureus.97863

**Published:** 2025-11-26

**Authors:** Juan Camilo Santacruz, Marta Juliana Mantilla, Sandra Pulido, Carlos Agudelo, Julián Alberto Naranjo, Oscar Vicente Vergara, John Londono

**Affiliations:** 1 Rheumatology, Medicarte IPS, Rionegro, COL; 2 Rheumatology, Centro de Investigación en Reumatología y Especialidades Médicas (CIREEM), Bogotá, COL; 3 Rheumatology, Colsanitas, Bogotá, COL; 4 Rheumatology, Clinica Las Americas, Medellín, COL; 5 Rheumatology, ART Médica IPS, Medellín, COL; 6 Spondyloarthropathies Research Group, Universidad de La Sabana, Chía, COL

**Keywords:** pulmonary hypertension, serositis, serum biomarkers, systemic lupus erythematosus, treatment choices

## Abstract

Pulmonary hypertension (PH) is an uncommon but serious manifestation of systemic lupus erythematosus (SLE) that significantly impacts prognosis. Its subtle clinical presentation often overlaps with other cardiopulmonary complications, leading to underdiagnosis and delayed treatment. Immune-mediated endothelial injury, chronic inflammation, and progressive vascular remodeling contribute to increased pulmonary arterial pressure and right ventricular dysfunction. Advances in molecular and immunologic research have identified distinct biological profiles in SLE-associated PH, highlighting the roles of endothelial dysfunction, cytokine imbalance, and specific autoantibodies in disease progression. These insights support precision medicine approaches targeting both immune and vascular pathways. Early recognition and multidisciplinary management are essential, with echocardiography and right heart catheterization remaining fundamental for diagnosis, and biomarkers aiding in risk stratification. Treatment typically combines immunosuppressive and pulmonary vasodilator therapies, with corticosteroids and cyclophosphamide as first-line options, while biologic agents and targeted vasodilator combinations may further enhance clinical outcomes. SLE-associated PH exemplifies the intersection of autoimmunity and vascular disease, and integrating novel diagnostic and therapeutic strategies may improve early detection and survival outcomes.

## Introduction and background

Systemic lupus erythematosus (SLE) is a chronic, multisystem autoimmune disease with a highly variable clinical course, typically marked by recurrent inflammatory flares over time. Its pathogenesis involves complex disturbances of the adaptive immune system, notably the generation of autoantibodies against nuclear antigens. These antibodies promote the formation and deposition of immune complexes, which can trigger tissue injury in virtually any organ or system [1]. A substantial proportion of patients develop pulmonary involvement during the course of their disease, affecting the lung parenchyma, pulmonary vasculature, pleura, and/or diaphragm [2].

Symptomatic pulmonary hypertension (PH) is considered an uncommon manifestation in SLE and often receives less clinical attention compared with its higher prevalence in systemic sclerosis and mixed connective tissue disease [3]. Reported prevalence in lupus varies widely, from 0.5% to 17.5% [4]. Approximately half of the cases appear idiopathic, while the remainder are secondary to advanced interstitial lung disease (ILD), pulmonary thromboembolism, pulmonary veno-occlusive disease, or cardiac involvement. Geographic differences are notable; in parts of Southeast Asia, SLE is the leading connective tissue disease associated with PH, with prevalence estimates ranging from 35% to 60% [5,6].

In a cross-sectional cohort of 283 lupus patients, Doppler echocardiography identified PH in 4% of individuals, with severe elevation of pulmonary artery systolic pressure (>40 mmHg) in about 1% [7]. Once PH is confirmed to be in World Symposium Group 1 (pulmonary arterial hypertension, PAH), patients are stratified into low-, intermediate-, or high-risk categories, with corresponding one-year mortality rates of <5%, 5-10%, and >10%, respectively [8]. Hemodynamically, PH is defined at right heart catheterization by a mean pulmonary arterial pressure (mPAP) >20 mmHg, pulmonary vascular resistance (PVR) >2 Wood units, and a pulmonary arterial wedge pressure ≤15 mmHg [9].

Clinical presentation varies: exertional dyspnea, fatigue, palpitations, and reduced exercise tolerance are common, while early disease may be asymptomatic. Other features can include syncope, weakness, peripheral edema, and abdominal distension. Raynaud’s phenomenon is reported in approximately 60% of cases [10]. Because PH is relatively infrequent in SLE, major guidelines do not recommend routine annual echocardiography or pulmonary function testing for screening purposes, which may lead to underdiagnosis and worse outcomes [11]. Consequently, a high index of suspicion is essential, combining risk factor assessment, biomarker evaluation, echocardiography, and full pulmonary function testing to enable timely diagnosis and management [12]. This review summarizes the epidemiologic patterns, clinical manifestations, pathophysiology, and therapeutic considerations of PH in SLE.

## Review

Epidemiology, classification, and risk factors

PH is categorized into five groups: (1) PAH, (2) PH due to left heart disease, (3) PH associated with lung disease or hypoxia, (4) PH caused by chronic thromboembolic or other pulmonary artery obstructions, and (5) PH with multifactorial or unclear mechanisms [13]. PAH may be idiopathic or secondary to various causes, including connective tissue diseases, exposure to drugs or toxins, or certain infections such as HIV, schistosomiasis, tuberculosis, human herpesvirus 8, and hepatitis C [14].

Several serological and clinical factors have been linked to PH development in lupus. A French cohort found anti-Sm, anti-SSA, and anti-SSB antibodies to be significant predictors [15]. In a Chinese cohort, serositis, anti-U1 RNP positivity, and a diffusing capacity for carbon monoxide (DLCO) <70% of predicted independently correlated with PH detection [15]. Across Western populations, pooled prevalence in lupus is around 13%, making it the second most frequent rheumatic cause after systemic sclerosis [16,17]. Observational studies have further associated digital vasculitis, Raynaud’s phenomenon, ILD, pericardial effusion, anti-U1 RNP antibodies, and anticardiolipin IgG with an elevated incidence of PH [18]. Interestingly, anti-U1 RNP positivity has been independently linked with greater PH severity, while pericardial effusion appears related to longer disease duration and worse prognosis [19].

Pathophysiology

PH is a vasculoproliferative disorder characterized by sustained vasoconstriction, increased vascular cell proliferation, fibrotic remodeling, and in situ microthrombosis [20]. Histopathologic examination typically shows thickening of the vessel wall with hyperplasia and hypertrophy of the intimal, medial, and adventitial layers, primarily affecting muscular pulmonary arterioles with diameters under 50 µm [21]. Fibrosis, small-vessel thrombosis, and advanced plexiform lesions are also common [22].

The vascular pathology in lupus-associated PH closely resembles that of idiopathic PAH, including endothelial and smooth muscle cell proliferation, fibrinoid necrosis, and deposition of immunoglobulins and immune complexes in the intima and media [23]. In some patients, plexiform lesions show inflammatory cell infiltrates composed mainly of lymphocytes and macrophages. Conversely, another subset exhibits a vasculopathic phenotype more akin to systemic sclerosis, with non-inflammatory vascular remodeling and occasional deposits of antiphospholipid antibodies, often in the context of anti-U1 RNP positivity [24,25].

Postmortem studies of pulmonary vessels in SLE patients with PH have demonstrated deposits of antinuclear antibodies, anti-double-stranded DNA, rheumatoid factor, immunoglobulins, and complement components - findings reminiscent of lupus nephritis. These immune deposits have been proposed as additional risk factors contributing to PH pathogenesis in SLE [26].

The exact mechanisms by which immune complexes in SLE can affect both large-caliber vessels and the pulmonary microvasculature remain incompletely understood [27]. Among the most representative manifestations of small-vessel injury are lupus pneumonitis and diffuse alveolar hemorrhage. Immune complex deposition has also been linked to the development of pulmonary veno-occlusive disease [27].

In addition to immune complex-mediated damage, increased levels of proinflammatory cytokines such as interleukin-1 (IL-1), IL-6, and tumor necrosis factor (TNF), as well as growth factors (A and B), and several chemokines including CCL2/monocyte chemoattractant protein-1 (MCP-1), RANTES/CCL5, and fractalkine/CX3CL1, have been described [28,29]. A study conducted in patients with SLE-associated PH identified the involvement of type I interferon (IFN) signaling, apoptosis, and protein ubiquitination in disease pathogenesis, along with aberrant T-cell activation promoting inflammation and vascular remodeling [30].

Histopathological studies have demonstrated infiltration of multiple immune cell types - including macrophages, dendritic cells, CD8+ and CD4+ T lymphocytes, and B cells - within the medial and adventitial layers of remodeled pulmonary vessels [31]. Alterations in the ubiquitin-proteasome system (UPS), which regulates protein degradation, gene expression, and apoptosis, have been associated with the proliferation of pulmonary arterial smooth muscle cells, suggesting that proteasome inhibitors may have therapeutic potential.

Furthermore, type I IFN, particularly IFN-α, appears to play a pivotal role in the pathogenesis of PH. In a murine model, the absence of functional IFNAR1 conferred protection against hypoxia-induced PH and vascular remodeling [32]. Collectively, these findings support a pathogenic link between type I IFN signaling and the development of PH in SLE, potentially mediated by endothelin-1 (ET-1), whose expression is induced by IFN [33].

Approximately 42% of patients with SLE-associated PH have autoantibodies targeting the endothelin type A receptor. These antibodies promote endothelial dysfunction and decrease prostacyclin synthesis - a key vasodilatory mediator - thereby contributing to increased PAP [34]. In SLE, elevated serum ET-1 concentrations correlate with IgM antibodies against vascular endothelial cells (AECAs) and with circulating immune complexes. This relationship suggests that IgM-AECAs may induce ET-1 release from endothelial cells, amplifying endothelial injury [35].

Although ILD is an uncommon and rarely severe cause of PH in SLE, two-thirds of patients may exhibit asymptomatic abnormalities on pulmonary function tests, and approximately one-third may present tomographic findings consistent with an ILD pattern [36]. Moreover, population-based data indicate that patients with SLE have a significantly higher risk of venous thromboembolic events, particularly in the presence of antiphospholipid syndrome [37].

**Figure 1:**
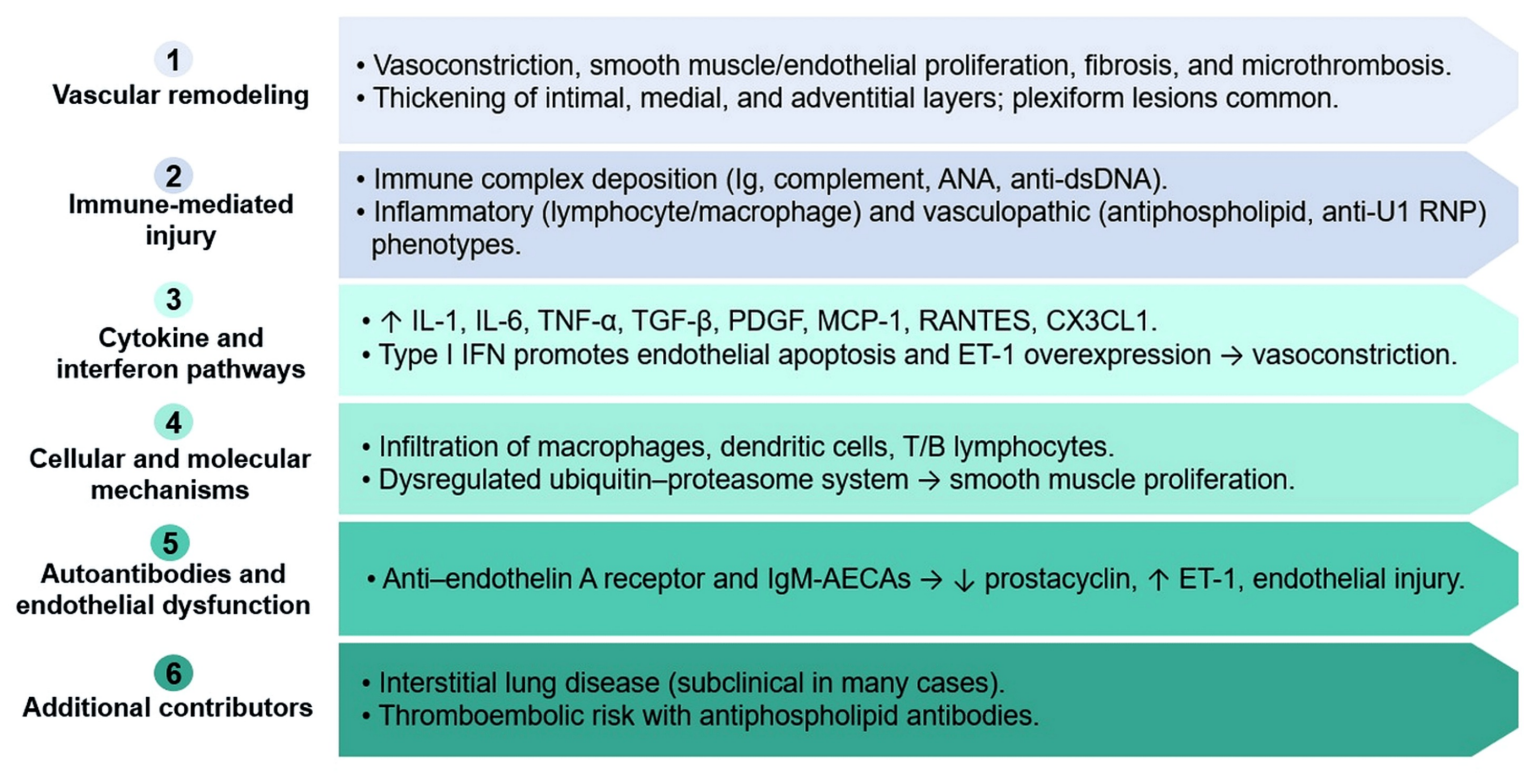
Proposed Pathogenic Mechanisms of Pulmonary Hypertension in Systemic Lupus Erythematosus ANA: antinuclear antibodies; anti-dsDNA: anti-double-stranded DNA antibodies; anti-U1 RNP: anti-U1 ribonucleoprotein antibodies; IL-1: interleukin-1; TNF-α: tumor necrosis factor-alpha; PDGF: platelet-derived growth factor; MCP-1: Monocyte chemoattractant protein-1; IFN: interferon; T/B: T lymphocytes/B lymphocytes; IgM-AECAs: immunoglobulin M anti-endothelial cell antibodies Credit: Image created by the authors.

A schematic summary of the key pathophysiological mechanisms involved in lupus-associated PH is presented in Figure 1.

Diagnosis

Screening for PH is justified when clinical predictors raise suspicion, particularly based on echocardiographic findings. Right heart catheterization remains the gold standard for diagnosing PAH. In patients with overlapping SLE and systemic sclerosis, or those classified under a cluster 3 phenotype, annual screening is recommended and should include transthoracic echocardiography, electrocardiography, biomarkers, pulmonary function tests, and cardiopulmonary exercise testing [38,39].

A tricuspid regurgitation velocity (TRV) ≥3.4 m/s indicates a high probability of PH, whereas a value between 2.9 and 3.4 m/s, when accompanied by signs of right ventricular overload, elevated pulmonary pressure, or right atrial enlargement, warrants further assessment through right heart catheterization [40]. Echocardiography also provides valuable information on biventricular morphology, ejection fraction, and valvular function.

Biomarkers such as brain natriuretic peptide (BNP) and N-terminal pro-BNP (NT-proBNP) can help identify SLE patients at high risk of PH. However, these peptides typically increase only when right ventricular dysfunction or significant PH progression is already present [41].

Right heart catheterization also enables assessment of vasoreactivity and helps determine the potential benefit of calcium channel blockers in PH management. Nevertheless, in a retrospective analysis of 663 patients with PH, of whom 168 had connective tissue disease, the acute vasodilator response and the long-term effect of calcium channel blockers were evaluated. While 10.1% demonstrated an acute positive response, only 0.6% were classified as long-term responders [42,43].

Stratification by means of biomarkers

Several studies have suggested that, beyond conventional diagnostic methods, certain biological markers may contribute to the detection of PH in the context of SLE, including antiphospholipid antibodies, anti-U1 RNP, ET-1, and uric acid [44]. In an observational study, circulating extracellular vesicles were isolated and characterized in patients with SLE, both with and without PH. A significant increase in platelet- and endothelium-derived vesicles, particularly annexin V-positive particles, was found in the PH group. These levels correlated with PAP and echocardiographic parameters, supporting their potential as non-invasive biomarkers [45].

Recent transcriptomic studies in SLE-associated PH have identified three molecular endotypes: one dominated by IFN/TNF signaling pathways linked to systemic inflammation; a second characterized by elevated IL-6 expression with a more benign hemodynamic profile; and a third defined by dysregulation of vascular smooth muscle genes, associated with greater disease severity and worse prognosis. These findings support a precision medicine approach targeting either immunomodulatory or vascular therapies according to the patient’s biological profile [46].

Autoantibodies directed against the ET-1 receptor have also been proposed as potential serum biomarkers with predictive and prognostic value in SLE-associated PH. In one study, these antibodies were detected in approximately 41.5% of patients with SLE and PH, compared to about 17% of patients with SLE without PH [47].

Multiple studies have demonstrated that the presence of IgG anticardiolipin antibodies and lupus anticoagulant is associated with an increased risk of developing PH in SLE, whereas other antiphospholipid antibodies did not show a significant association [48]. The CSTAR cohort, which included 3,624 patients with SLE, identified anti-RNP and anti-Ro/La antibodies as significant predictors of PH in this setting. Similarly, a smaller cohort of 74 SLE patients showed a higher frequency of rheumatoid factor positivity among those with PH compared to those without. Additional serologic risk markers for PH development include anti-Scl-70, anti-Sm positivity, low erythrocyte sedimentation rate, and hypocomplementemia [49].

In a cross-sectional cohort study involving 114 SLE patients, those with PH exhibited higher uric acid levels compared to patients without PH. Furthermore, a direct correlation was observed between serum uric acid concentrations and PAP [50]. Sustained uric acid levels ≥7 mg/dL in patients with initially normal pulmonary artery pressure have been associated with an increased risk of developing PH over a follow-up period of up to seven years, positioning uric acid as one of the most promising biomarkers for identifying patients at risk during early stages [51].

Treatment

It is strongly recommended that all patients with SLE-associated PH be evaluated at specialized PH centers [52]. Supportive care should be individualized based on clinical status and may include supplemental oxygen, light physical activity, and diuretics in the presence of right heart failure. Immunosuppressive therapy, particularly the combination of glucocorticoids and cyclophosphamide, has shown significant improvement in hemodynamic parameters when initiated early [53]. Glucocorticoids - most often prednisolone or intravenous methylprednisolone pulses (500 mg to 1 g daily for three days) - are typically administered alongside intravenous cyclophosphamide at 0.5-1 g/m^2^ monthly, producing both clinical and functional benefits [54]. In Luo et al., 61% of patients achieved hemodynamic and functional improvement when combined with vasodilators such as bosentan or sildenafil [54].

For cyclophosphamide-refractory cases, rituximab has demonstrated clinical benefit, with improvements in New York Heart Association (NYHA) functional class, mean pulmonary artery pressure (from 55 to 39 mmHg), BNP levels, and six-minute walk distance [55]. In moderate-to-severe disease, adjunctive vasodilator therapy is recommended. Combination therapy with ambrisentan and tadalafil reduced the risk of clinical worsening compared to monotherapy in the AMBITION trial, which included patients with connective tissue disease and lupus [56,57]. Sildenafil monotherapy (20 mg orally every eight hours) improved exercise capacity and hemodynamics in a smaller series [58].

Prostacyclin pathway agonists, such as intravenous epoprostenol, treprostinil (inhaled, subcutaneous, or intravenous), inhaled iloprost, and selexipag, are key options for advanced cases or suboptimal responders [59]. Long-term intravenous epoprostenol (4-46 ng/kg/min) has produced marked hemodynamic improvement in SLE-PH [60]. Subcutaneous treprostinil (mean dose 8.4 ng/kg/min at 12 weeks) improved exercise tolerance, dyspnea, and fatigue [61]. More recently, sotatercept, an activin signaling inhibitor, demonstrated clinical and hemodynamic benefit in mild-to-moderate PH in the STELLAR trial and in smaller cohorts of connective tissue disease-associated PH, even in those with ILD [62,63].

**Figure 2:**
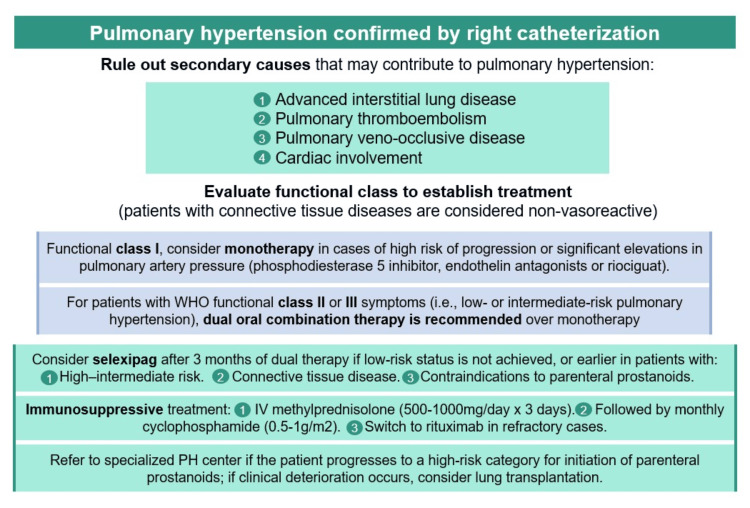
Algorithm for the Evaluation and Management of Pulmonary Hypertension in Patients With Systemic Lupus Erythematosus Credit: Image created by the authors.

The following section presents a comprehensive table summarizing the main risk factors and biomarkers associated with the pathogenesis of PH in SLE, along with a treatment algorithm developed according to the current levels of evidence (Table 1 and Figure 2).

**Table 1:** Main Risk Factors and Biomarkers Associated With Pulmonary Hypertension in Systemic Lupus Erythematosus DLCO: diffusing capacity for carbon monoxide; ET-1: endothelin-1; ETAR: endothelin type A receptor; ESR: erythrocyte sedimentation rate; IFN: interferon; IgG: immunoglobulin G; IgM: immunoglobulin M; ILD: interstitial lung disease; IL-6: interleukin-6; PH: pulmonary hypertension; RF: rheumatoid factor; RNP: ribonucleoprotein; Scl-70: topoisomerase I antibody; SLE: systemic lupus erythematosus; Sm: Smith antibody; SSA (Ro): Sjögren’s-syndrome-related antigen A

Category	Risk Factor/Biomarker	References
Clinical features	Raynaud’s phenomenon, digital vasculitis, serositis, interstitial lung disease, pericardial effusion, and longer disease duration are associated with higher PH risk and worse prognosis	[15,18,19]
Autoantibodies	Anti-U1 RNP (predictor of PH and associated with greater severity)	[15,19,49]
	Anti-Sm, anti-SSA (Ro), and anti-SSB (La) antibodies are associated with PH development	[15,49]
	Anticardiolipin IgG and lupus anticoagulant correlated with higher PH risk	[48]
	Anti-endothelin type A receptor antibodies (present in ~41.5% of SLE-PH vs. 17% of SLE without PH)	[47]
	Rheumatoid factor positivity linked to PH presence in smaller cohorts	[49]
	Anti-Scl-70 and anti-Sm antibodies are associated with an increased risk of PH	[49]
Complement and inflammatory markers	Hypocomplementemia and low ESR are associated with PH risk	[49]
Cytokines and molecular pathways	Type I IFN/TNF and IL-6 transcriptomic signatures define molecular endotypes with distinct hemodynamic and prognostic profiles	[46]
Endothelin system	ET-1 levels correlated with endothelial injury and the presence of circulating IgM anti-endothelial cell antibodies	[35]
Extracellular vesicles	Increased platelet- and endothelium-derived extracellular vesicles (Annexin V+) correlate with pulmonary arterial pressure and echocardiographic findings	[45]
Metabolic biomarkers	Elevated serum uric acid (≥7 mg/dL) predicts future PH development during follow-up (up to seven years)	[50,51]
Pulmonary function	DLCO <70% predicted is independently associated with PH detection	[15]
Antiphospholipid syndrome	Coexistence increases the risk of thromboembolic PH and a worse hemodynamic profile	[37,48]

Prognosis

There is limited data regarding the survival of patients with SLE-associated PH. The largest retrospective cohort, which included 51 patients, reported three- and five-year survival rates of 89.4% and 83.9%, respectively. The mean time to diagnosis was approximately 4.9 years, and the presence of anti-U1 RNP antibodies was identified as a predictor of better outcomes [64]. A systematic review found that higher functional class (III/IV), elevated mPAP and PVR, shorter six-minute walk distance, and increased BNP/NT-proBNP levels were associated with poorer survival [65]. Predominantly vasculopathic features, particularly Raynaud’s phenomenon, appear to be associated with improved survival and better treatment response [66].

## Conclusions

PH associated with SLE represents a complex manifestation in which immune and vascular mechanisms converge, significantly impacting morbidity and mortality. Its pathophysiology involves endothelial dysfunction, platelet activation, and proinflammatory cytokine networks, particularly those mediated by IFN and TNF, that promote vascular remodeling. The identification of molecular profiles and potential biomarkers, such as endothelial autoantibodies or elevated uric acid levels, opens opportunities for earlier and more targeted diagnostic and therapeutic approaches. A combination of immunosuppressive and vasodilator therapies, tailored to the clinical and immunological phenotype, appears to be the most promising strategy to modify disease progression. In this context, the integration of precision medicine tools and close multidisciplinary collaboration between rheumatology, cardiology, and pulmonology are essential to optimize clinical outcomes and improve survival in patients with this severe lupus complication.
